# RBM10 inhibits cell proliferation of lung adenocarcinoma via RAP1/AKT/CREB signalling pathway

**DOI:** 10.1111/jcmm.14263

**Published:** 2019-04-06

**Authors:** Xin Jin, Xin Di, Ruimin Wang, He Ma, Chang Tian, Min Zhao, Shan Cong, Jiaying Liu, Ranwei Li, Ke Wang

**Affiliations:** ^1^ Department of Respiratory Medicine The Second Hospital of Jilin University Changchun, Jilin China; ^2^ Department of Oncology and Hematology The Second Hospital of Jilin University Changchun, Jilin China; ^3^ Department of Operation room The Second Hospital of Jilin University Changchun, Jilin China; ^4^ Department of Anesthesiology The Second Hospital of Jilin University Changchun, Jilin China; ^5^ Department of Urinary Surgery The Second Hospital of Jilin University Changchun, Jilin China

**Keywords:** AKT, CREB, lung cancer, proliferation, RAP1, RNA‐binding motif protein 10

## Abstract

Initial functional studies have demonstrated that RNA‐binding motif protein 10 (RBM10) can promote apoptosis and suppress cell proliferation; however, the results of several studies suggest a tumour‐promoting role for RBM10. Herein, we assessed the involvement of RBM10 in lung adenocarcinoma cell proliferation and explored the potential molecular mechanism. We found that, both in vitro and in vivo, RBM10 overexpression suppresses lung adenocarcinoma cell proliferation, while its knockdown enhances cell proliferation. Using complementary DNA microarray analysis, we previously found that RBM10 overexpression induces significant down‐regulation of RAP1A expression. In this study, we have confirmed that RBM10 decreases the activation of RAP1 and found that EPAC stimulation and inhibition can abolish the effects of RBM10 knockdown and overexpression, respectively, and regulate cell growth. This effect of RBM10 on proliferation was independent of the MAPK/ERK and P38/MAPK signalling pathways. We found that RBM10 reduces the phosphorylation of CREB via the AKT signalling pathway, suggesting that RBM10 exhibits its effect on lung adenocarcinoma cell proliferation via the RAP1/AKT/CREB signalling pathway.

## 1 INTRODUCTION

Lung cancer is the most commonly diagnosed cancer (11.6%) and the leading cause of cancer‐caused death (18.4%).^1^ For purposes of treatment, lung cancer is classified either as small cell lung cancer (13%) or non‐small cell lung cancer (NSCLC; 87%), for which the overall 5‐year survival rate is only 18.2%.^2^ In recent years, molecular targeted therapies have dramatically improved progression‐free survival and overall survival for NSCLC patients with activated oncogenes such as mutant EGFR or translocated ALK,RET, or ROS1^3^, ^4^ and are currently the most promising approach for treating NSCLC patients. The Cancer Genome Atlas Research Network found that EGFR mutations are more frequent among female lung adenocarcinoma patients, whereas mutations in RNA‐binding motif protein 10 (RBM10) are more common among male patients.^7^

RBM10 is a member of the RNA‐binding protein (RBP) family, is located at position p11.23 of the X‐chromosome, and is known for its role in mRNA splicing.^8^ Initial functional studies have demonstrated that RBM10 can promote apoptosis^9^ and suppress cell proliferation.^10^, ^11^ Furthermore, its expression was found to be correlated with increased expression of the proapoptotic protein BAX and the tumour suppressor protein TP53 in breast cancer.^13^ These findings suggest RBM10 as a potential tumour suppressor. However, insufficient research has focused on RBM10‐related signalling pathways, which would explain the mechanism of its tumour‐suppressing effect.

To elucidate the underlying molecular mechanism, we previously performed an Affymetrix GeneChip Primeview Human cDNA microarray analysis. A total of 690 genes that were regulated by RBM10 expression in A549 cells were identified, of which 304 were up‐regulated and 386 were down‐regulated. Among these, RAP1A expression was the most down‐regulated (63.5%)^14^; thus, we explored its downstream signalling pathway. RAP1A is a type of Ras‐associated protein 1 (RAP1), with 95% sequence homology to RAP1B.^15^ It is a significant regulator and mediator of Ras functions, and its activation has been linked to a variety of cancers.^16^, ^17^ EPAC is a guanine nucleotide exchange factor (GEF) for the RAP1, which activates RAP1 by GDP‐GTP exchange.^21^, ^22^ Agonists and antagonists selective for EPAC have been developed and can be used for further studies on the activation of RAP1, which will increase our understanding on the signalling pathway.^23^

In this study, we aimed to explore the effect of RBM10 overexpression and knockdown on the proliferation of lung adenocarcinoma cells as well as the affected downstream pathways by assessing the expression of several regulatory proteins that have previously been identified as being modulated by RBM10.^14^ We then sought to verify the effect of RBM10 overexpression and inhibition in vivo in BALB/c mice.

## 2 MATERIALS AND METHODS

### 2.1 Cell culture and reagents

The human NSCLC cell lines A549 and H1299 were obtained from the Chinese Academy of Medical Sciences (Beijing, China) and cultured in RPMI1640 (Invitrogen, Carlsbad, CA) supplemented with 10% foetal bovine serum and 100 U/mL penicillin and 0.1 mg/mL streptomycin (Invitrogen) at 37°C, in a humidified atmosphere containing 5% CO_2_. Dimethylsulfoxide (DMSO) was purchased from Sigma‐Aldrich (St. Louis, MO) 0.8‐pCPT‐2’‐OMe‐cAMP and ESI‐09 were obtained from the Biological Life Science Institute (Bremen, Germany).

### 2.2 Vector construction and cell transfection

The lentiviral vectorsGV358‐RBM10 (14297‐1; Ubi‐MCS‐3FLAG‐SV40‐EGFP‐IRES‐puromycin) and GV248‐RBM10‐RNAi (66648‐1;hU6‐shTDP‐43‐Ubi‐EGFP‐IRES‐puromycin) and the corresponding control lentiviruses, CON238 and CON077, respectively, were obtained from Genechem (Shanghai, China). A549 cells and H1299 cells were infected with these lentiviral vectors. A total of 5 × 10^5^ A549 cells and H1299 cells was seeded in a six‐well cell plate and further incubated for 12 hours to reach 30% confluency. A549 cells were infected with the lentiviral vectors at a multiplicity of infection (MOI) of 20 plaque‐forming units (PFU) per cell and H1299 cells at a MOI of 5 PFU per cell. The plates were then incubated for 24 hours prior to having their media changed to fresh, virus‐free media. Three days later, the GFP density was examined to evaluate the lentiviral infection efficiency.

### 2.3 Cell viability assay

Cell Counting Kit 8 (CCK‐8) was employed to determine the viability of infected cells (2000 cells/well) that were seeded in 96‐well plates in RPMI1640. After 24, 48, 72 and 96 hours, 10 μL of CCK‐8 was added into each well and incubated for 1 hours. Absorbance at 450 nm was read using a microplate reader (Bio‐Rad, Hercules, CA). Data were calculated from three independent experiments, and each was conducted in triplicate.

### 2.4 Colony formation assay

A549 and H1299 cells were seeded in six‐well plates at a density of 500 cells per well and cultured in RPMI 1640 containing 10% of FBS for 2 weeks. Cells were then fixed with 4% paraformaldehyde for 10 min, stained with Giemsa for 10 min and washed three times with ddH_2_O. The cells were photographed with a digital camera, and colonies of more than 50 cells were counted. The assay was performed in triplicate and repeated at least twice.

### 2.5 Western blotting assay

Protein concentrations in the supernatants were quantified using the DC Protein Assay (Bio‐Rad), and 50 μg aliquots of each protein sample were added to the sample buffer (10% glycerol, 0.7 mol/L beta‐mercaptoethanol, 3% sodium dodecyl sulfate, 62 mmol/L Tris‐HCl, pH 6.8), boiled for 10 min, separated using 12% sodium dodecyl sulfate‐polyacrylamide gel electrophoresis and then transferred onto polyvinylidene fluoride membranes (Millipore, Billerica, MA). For Western blotting, membranes were incubated in 50 g/L skimmed non‐fat milk/Tris‐buffered saline and Tween‐20 solution (TBST) at room temperature for 1 hour and then incubated overnight at 4 ℃ with a primary antibody. The following primary antibodies were used in this study: RBM10 (#14423‐1‐AP; 1:500), β‐actin (#60008‐1‐Ig; 1:2000), β‐tubulin (#66240‐1‐Ig; 1:20000) from Proteintech Group (Chicago, IL, USA); RAP1 (#8818; 1:1000), phospho‐ERK1/2 (Thr202/Tyr204;#4370; 1:1000), ERK1/2 (#4695; 1:1000), phospho‐P38 MAPK (Thr180/Tyr182;#4511; 1:1000), P38 MAPK (#8690; 1:1000), phospho‐AKT(Ser473;#4060; 1:2000), phospho‐AKT(Thr308;#13038; 1:1000) and AKT (#4691; 1:1000) from Cell Signalling Technology (Danvers, MA, USA); and CREB (phospho‐S133; #ab32096; 1:5000) and CREB (#ab32515; 1:1000) from Abcam (Cambridge, MA). The next day, membranes were washed with TBST three times for 10 min each and then further incubated with corresponding horseradish peroxidase‐conjugated secondary antibody (#SA00001‐1 or #SA00001‐2; Proteintech Group) at a dilution of 1:2000 to 1:5000 for 1 hour at room temperature. After washing the membranes three times for 10 min each with TBST, protein bands were detected using Amersham ECL Plus Western Blotting Detection Reagent (Millipore) and quantified using Quantity One software (Bio‐Rad). The reproducibility of the experiments was tested by repeating them at least three times.

### 2.6 RAP1‐GTP pull‐down assay

Cells were lysed with radioimmunoprecipitation assay (RIPA) buffer with protease and phosphatase inhibitors. An equal amount of protein (approximately 150 mg) was incubated with GST‐RAP‐binding domain (RBD) of RalGDS conjugated to glutathione beads for 2 hour at 4°C and centrifuged for 30 s. Beads were washed with RIPA buffer and mixed with loading dye containing β‐mercaptoethanol. RAP1‐GTP levels were detected by Western blotting.

### 2.7 Xenograft experiment

The use of animals in this study was in accordance with animal care guidelines, and the protocol was approved by the Jilin University Animal Care Committee. A549 xenografts were established and the RBM10 gene was delivered into the xenografts by attenuated Salmonella. Briefly, BALB/c athymic nude male mice (nu/nu; between 4 and 5 weeks old) were purchased from Beijing Vital River Laboratory Animal Technology (Beijing, China). The mice were randomly placed into four groups of six each, and the backs of the mice were labeled with picric acid at different locations. A549 cells (1 × 10^7^) were suspended in 100 μL PBS and injected subcutaneously into the left armpit region of the nude mice. After 27 days, the mice were sacrificed, the explants were excised and images were captured.

### 2.8 Statistical analysis

Data were analysed by SPSS 24 software (IBM, Chicago, IL) and are expressed as means ± SD. Analysis of significant differences between groups was performed using unpaired Student's *t* test or one‐way ANOVA *P* < 0.05 was considered as a statistically significant difference.

## 3 RESULTS

### 3.1 RBM10 overexpression suppresses cell proliferation, while RBM10 knockdown enhances cell proliferation in vitro

**Figure 1:**
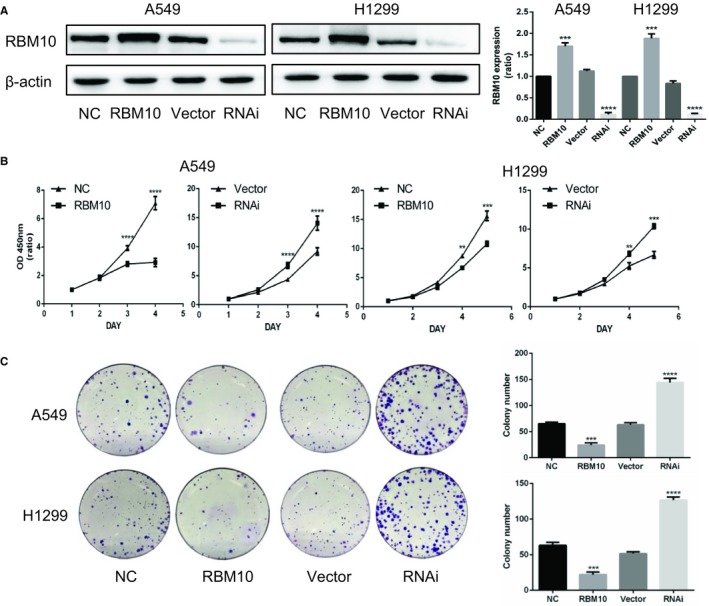
RBM10 inhibits proliferation of A549 and H1299 cells. A, A549 and H1299 cells were transfected with lentiviruses expressing GV358‐RBM10 (RBM10) and GV248‐RBM10‐RNAi (RNAi) and with their corresponding controls, GV358 (NC) and GV248 (Vector), respectively. Total protein extracts from the cells were analysed by Western blotting for RBM10 expression. B, The effect of RBM10 on cell viability was measured by the CCK‐8 assay. C, Stably transfected cells were subjected to colony formation assays and incubated for 14 d. Colonies were photographed and counted. **P < 0.01, ***P < 0.001, ****P < 0.0001. Data represent mean values ± SD

We first performed functional in vitro assays to explore the involvement of RBM10 in NSCLC progression. RBM10 was either overexpressed or silenced in A549 and H1299 cells through lentiviral transduction. The expression efficiency was determined by Western blotting (Figure 1A). Results of CCK‐8 and colony formation assays indicated that RBM10 overexpression significantly attenuated the cell proliferation ability, whereas its knockdown markedly enhanced cell proliferation (Figure 1B,C).These results indicate that RBM10 overexpression could suppress and RBM10 knockdown could enhance cell growth in vitro.

### 3.2 RBM10 inhibits cell proliferation by decreasing the activation of RAP1, and EPAC stimulation or inhibition can reverse it

**Figure 2:**
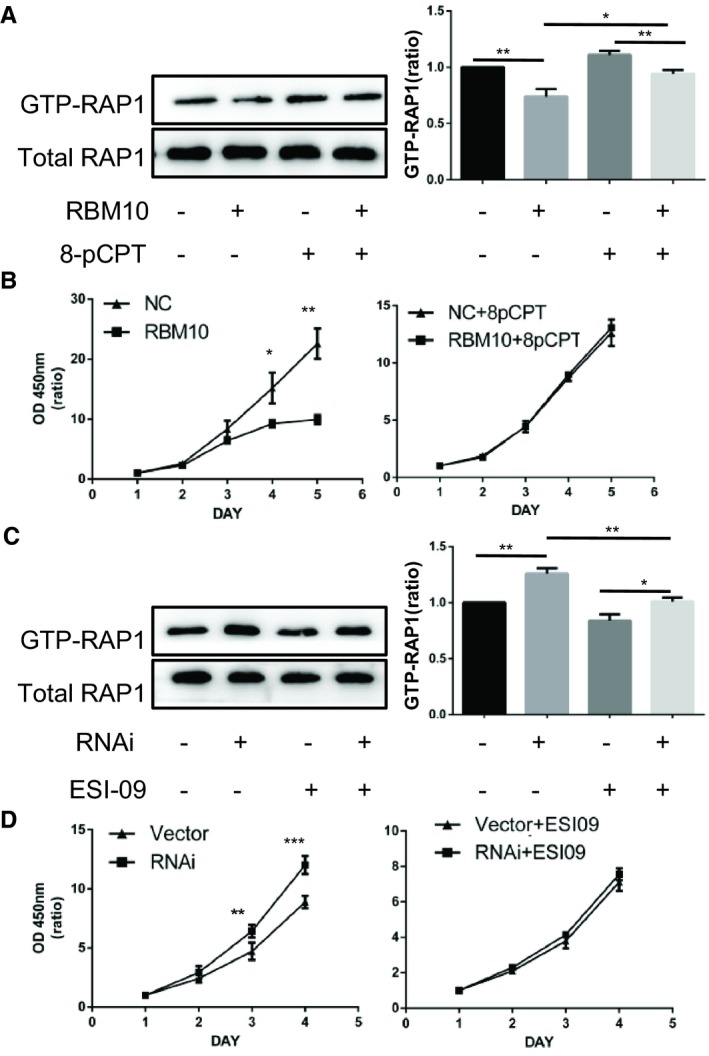
RBM10 decreases the activation of RAP1. A, A549 cells were transfected with GV358‐RBM10 and with its corresponding control vectors (NC) and subjected to pull‐down assay using GST‐RBD, followed by immunoblotting with antibodies against RAP1; GV358‐RBM10 and NC were treated with PBS (P) or 100 μmol/L of the EPAC stimulator 8‐pCPT‐2’‐O‐Me‐cAMP (8‐pCPT) for 30 min, and GTP‐RAP1 was detected by western blotting. B: The effect of RBM10 overexpression on cell viability was measured by the CCK‐8 assay. C: A549 cells were transfected with GV248‐RBM10‐RNAi and with its corresponding control vector (V) subjected to pull‐down assay using GST‐RBD followed by immunoblotting with antibodies to RAP1; GV248‐RBM10‐RNAi and Vector were treated with DMSO (D) or 10 μmol/L of the EPAC inhibitor ESI‐09 for 30 min, and GTP‐RAP1 was detected by Western blotting. D: The effect of RBM10 knockdown on cell viability was measured by the CCK‐8 assay. *P < 0.05, **P < 0.01, ***P < 0.001. Data represent mean values ± SD

The activation of RAP1, a well‐known EPAC effector, previously selected by the microarray analysis, was examined by Western blotting (Figure 2A,C). Next, the effect of the EPAC stimulator 8‐pCPT‐2’‐O‐Me‐cAMP and inhibitor ESI‐09 was examined. Treatment with 8‐pCPT‐2’‐O‐Me‐cAMP increased GTP‐bound RAP1 expression, which indicated increased RAP1 activation (Figure 2A). On the other hand, EPAC inhibition using ESI‐09 abolished the RBM10‐RNAi‐induced increase in GTP‐bound RAP1 expression (Figure 2C). Results of the CCK‐8 assay indicated that 8‐pCPT‐2’‐O‐Me‐cAMP significantly reversed the inhibition of cell growth in RBM10‐overexpressing A549 cells and that ESI‐09 significantly decreased cell proliferation in RBM10‐RNAi‐treated cells (Figure 2B,D). These results indicate that RBM10 inhibits cell proliferation by decreasing the expression of GTP‐bound RAP1 via the EPAC/RAP1‐mediated pathway.

### 3.3 RBM10‐mediated cell proliferation is independent of MAPK/ERK and P38 MAPK signalling pathways

**Figure 3:**
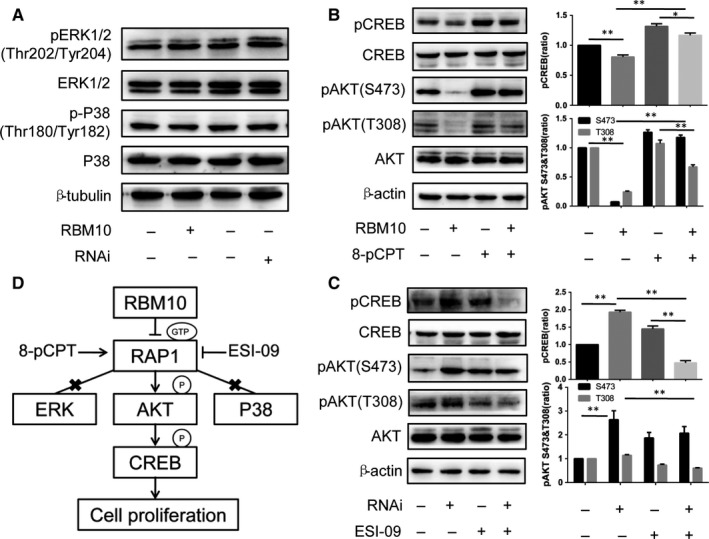
Effect of RBM10 on the MAPK/ERK, P38 MAPK and AKT/CREB signalling pathways. A, The effect of RBM10 on ERK and P38 distribution in A549 cells was examined by western blotting. B, The effect of GV358‐RBM10 and treatment with the EPAC stimulator 8‐pCPT‐2’‐O‐Me‐cAMP on phospho‐AKT and phospho‐CREB distribution in A549 cells was examined by western blotting. C: The effect of GV248‐RBM10‐RNAi and treatment with the EPAC inhibitor ESI‐09 on phospho‐AKT and phospho‐CREB distribution in A549 cells was examined by Western blotting. D: A model for the role of RBM10 in lung adenocarcinoma cell proliferation. RBM10 inhibits proliferation via the RAP1/AKT/CREB signalling pathway. *P < 0.05, **P < 0.01. Data represent mean values ± SD

To determine whether the effects of RBM10 on cell proliferation were associated with the MAPK/ERK or P38 MAPK pathways, we assessed the expression of key markers of these pathways that are closely associated with GTP‐bound RAP1 expression. Compared to the control group, no significant changes in the expression of phospho‐ERK1/2 (Thr202/Tyr204) and phospho‐P38 MAPK (Thr180/Tyr182) were found in RBM10‐overexpressing and ‐knockdown cells (Figure 3A). These results suggest that RBM10‐mediated regulation of cell proliferation is not dependent on the MAPK/ERK and P38 MAPK signalling pathways.

### 3.4 RBM10 reduces the phosphorylation of CREB via AKT signalling pathway

Overexpression of RBM10 resulted in decreased phospho‐AKT and phospho‐CREB in A549 cells (Figure 3B). In contrast, the expression of phospho‐AKT and phospho‐CREB in RBM10‐knockdown A549 cells was increased greatly compared to the control group (Figure 3C). Then, treatment with the EPAC stimulator 8‐pCPT‐2’‐O‐Me‐cAMP and inhibitor ESI‐09 reversed the expression of phospho‐AKT and phospho‐CREB (Figure 3B,C). Together with the results presented above, these findings suggest that RBM10 suppresses the phosphorylation of AKT and consequently decreases the expression of CREB, which is involved in the regulation of cell proliferation in lung adenocarcinoma cells.

### 3.5 RBM10 inhibits lung tumour growth in vivo

**Figure 4:**
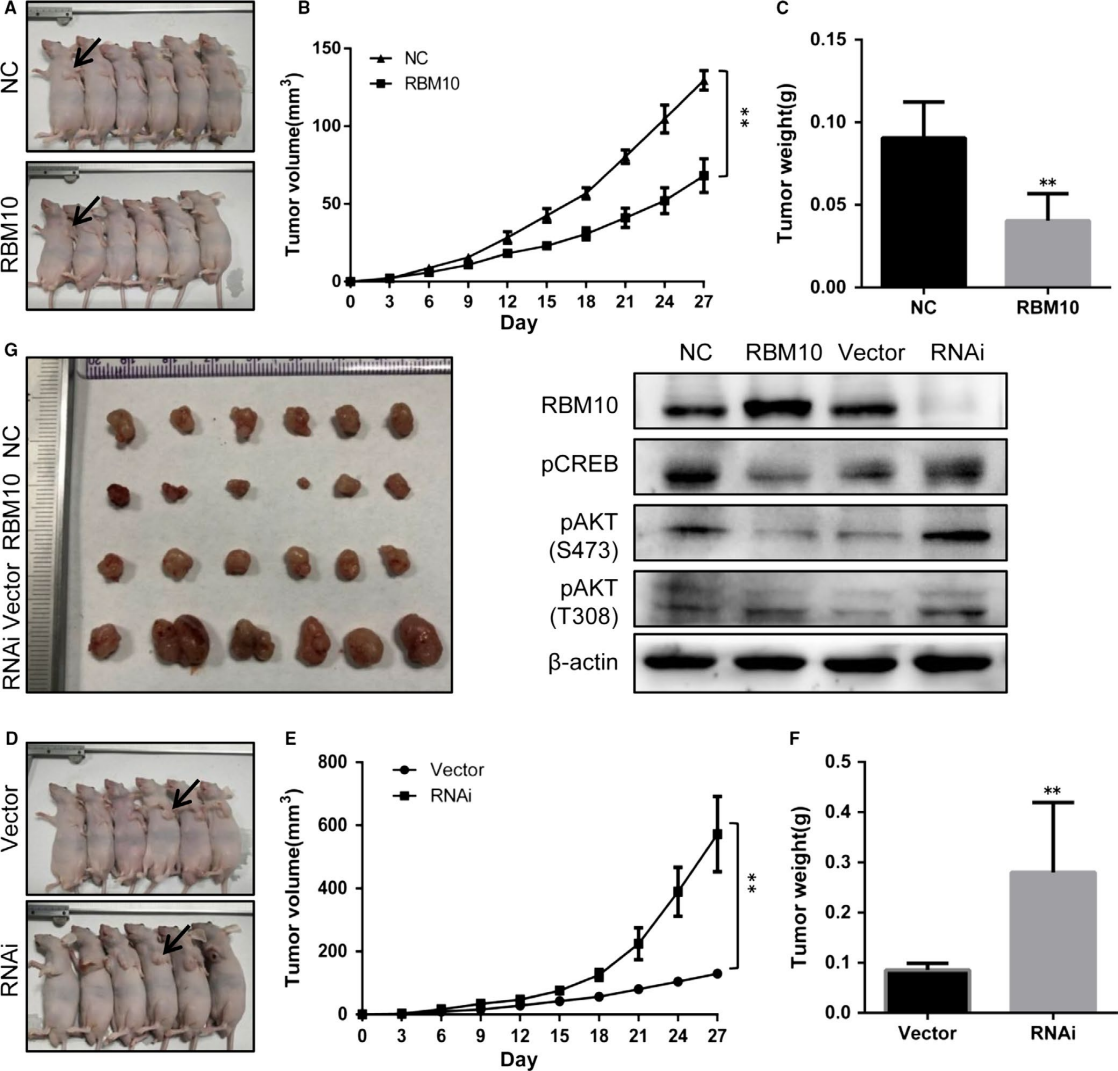
RBM10 inhibits lung tumour growth in vivo. A, A549 cells overexpressing RBM10 were subcutaneously injected into nude mice, and the mice were monitored for 27 d. A representative picture of tumours thus formed is shown. B, A growth curve of tumour volumes was constructed every 3 d for 27 d. C, Tumour weight was measured. D, A549 cells expressing RBM10‐RNAi were subcutaneously injected into nude mice, and the mice were monitored for 27 d. A representative picture of tumours thus formed is shown. E, A growth curve of tumour volumes was constructed every 3 d for 27 d. F, Tumour weight was measured. G, Photograph shows the dissected tumours formed by the inoculated infectants. H, Western blotting analyses of the expression of phospho‐AKT and phospho‐CREB in tumour tissues. **P < 0.01. Data represent mean values ± SD

To verify the in vitro observation of the antitumour effect of RBM10 overexpression, a murine in vivo xenograft model of lung adenocarcinoma was established. A549 cells infected with the GV358‐RBM10 lentivirus, causing overexpression of RBM10, or negative control cells were injected into nude mice and the tumour growth activity was measured. Compared to the control, the average tumour volume (Figure 4A,B,G) and weight (Figure 4C) of the GV358‐RBM10‐treated group was markedly lower. On the other hand, infection with the GV248‐RBM10‐RNAi lentivirus, silencing RBM10 expression, resulted in accelerated xenograft tumour growth compared to the control group treated with the vector only(Figure 4D‐G). Western blotting revealed that the expression of phospho‐AKT and phospho‐CREB was remarkably changed in RBM10‐inoculated tumour tissues (Figure 4H).These results indicate that RBM10 functions as a tumour‐suppressing molecule and negatively regulates lung adenocarcinoma cell growth in vivo.

## 4 DISCUSSION

According to a recent review, results suggesting a tumour‐promoting role for RBM10 have emerged.^24^ Several recent findings support this: (a) RBM10 expression is correlated with increased mRNA expression of VEGF, a potent angiogenic promoter^13^; (b) knockdown of RBM10 expression is predicted to reduce epithelial to mesenchymal transition through the association of RBM10 with FilGAP, a regulator of cell migration^25^; (c)in the endogenous RBM5‐null GLC20 small cell lung cancer cell line, RBM10 promoted cell proliferation and other transformation‐associated processes.^26^ To clarify the role of RBM10 in lung adenocarcinoma, we designed a 2‐fold(overexpression and knockdown) study of RBM10. Our results confirmed that its overexpression suppresses cell proliferation and its knockdown enhances cell growth both in vitro and in vivo. These results indicate that RBM10 plays a role in tumour suppression in lung adenocarcinoma.

To elucidate the molecular mechanism behind this finding, we previously discovered that RAP1A expression was the most down‐regulated in the cDNA microarray analysis and we explored its downstream signalling pathways. Among them, the synergistic activation of the ERK signalling pathway is the most common related pathway.^27^, ^28^ However, in this study, there was no change in the phosphorylation of ERK after RAP1 activation. Similarly, the P38 MAPK signalling pathway has been reported to be involved in the activation of RAP1,^30^ but we observed no change in P38 phosphorylation after RAP1 activation. Finally, we found that overexpression of RBM10 resulted in decreased phospho‐AKT levels, while RBM10 silencing induced up‐regulated phospho‐AKT expression.

To further explore the effect of activated RAP1 on AKT phosphorylation, the EPAC stimulator 8‐pCPT‐2’‐O‐Me‐cAMP and inhibitor ESI‐09 were applied after RBM10 overexpression or knockdown in A549 cells. The result indicated that the activation of EPAC signalling with 8‐pCPT‐2’‐O‐Me‐cAMP induced the activating phosphorylation of AKT, while ESI‐09 restored AKT phosphorylation induced by RBM10 knockdown. These results showed that RBM10‐mediated cell proliferation is dependent on the AKT signalling pathway.

The CREB protein is a crucial transcription factor that regulates a wide range of biological processes that orchestrate cell differentiation and cell growth.^31^ CREB enhances cell proliferation, reduces apoptosis sensitivity and increases angiogenesis and radiation‐induced differentiation.^32^ Overexpression of CREB was reported in many solid tumour types like breast cancer, liver cancer and melanoma when compared to adjacent normal tissues.^33^, ^34^ CREB is an important factor in cAMP signalling, which regulates protein levels by controlling gene transcription. cAMP signalling commonly activates three major cAMP effector molecules: PKA, EPAC and cyclic‐nucleotide‐gated ion channels.^36^ Herein, we revealed that RBM10 mediates an increase in CREB expression and found that EPAC/RAP1, but not PKA, mediates CREB expression. This result is further supported by the observation that CREB expression increased after EPAC‐selective stimulation and decreased after EPAC‐selective inhibition. In conclusion, RBM10 inhibited the expression of CREB via EPAC/RAP1‐specific signalling.

To conclude, this study found that RBM10 inhibits lung adenocarcinoma cell growth in vitro and in vivo. Specifically, it inhibits cell proliferation via the RAP1/AKT/CREB signalling pathway. Our observations may help to better understand how deregulation of RBM10 contributes to lung adenocarcinoma progression.

## CONFLICT OF INTEREST

The authors confirm that there are no conflict of interest.
